# Effect of Ti_3_C_2_T_*x*_ MXenes etched at elevated temperatures using concentrated acid on binder-free supercapacitors†

**DOI:** 10.1039/d0ra05376g

**Published:** 2020-11-17

**Authors:** Sunil Kumar, Dongwoon Kang, Hyeryeon Hong, Malik Abdul Rehman, Yeon-jae Lee, Naesung Lee, Yongho Seo

**Affiliations:** Department of Nanotechnology and Advanced Materials Engineering, Sejong University Seoul 05006 South Korea yseo@sejong.ac.kr; HMC, Sejong University Seoul 05006 South Korea; Department of Materials Science and Engineering, Yonsei University Seoul 03722 South Korea

## Abstract

The effect of Ti_3_C_2_T_*x*_ MXene etched at different temperatures (25 °C, 50 °C, and 80 °C) on the capacitance of supercapacitors without the use of conducting carbon-black or a binder was studied. The MXene etched using concentrated HCl acid (12 M)/LiF was used as an active electrode and Ni-foil as a current collector. It was observed that the elevated etching temperature facilitates the etching of the MAX phase and the exfoliation of MXene layers. However, this led to the formation of additional functional groups at the MXene surface as the temperature was increased to 80 °C. The specific capacitance of Ti_3_C_2_T_*x*_-based supercapacitors increased from 581 F g^−1^ for MXene etched at 25 °C to 657 F g^−1^ for those etched at 50 °C at the scan rate of 2 mV s^−1^. However, the specific capacitance reduced to 421 F g^−1^ as the etching temperature was increased to 80 °C at the same scan rate. The supercapacitors based on MXenes etched at the intermediate temperature (50 °C) exhibited higher specific capacitance in a wide range of scan rate, symmetry in charge/discharge curves, high cyclic stability at a scan rate of 1000 mV s^−1^ for up to 3000 cycles. The electrochemical impedance spectroscopy studies indicated low series resistance, reduced charge-transfer resistance, and decreased Warburg impedance for the supercapacitor based on the MXene etched at the intermediate temperature.

## Abstract 2

The effect of Ti_3_C_2_T_*x*_ MXene etched at different temperatures (25 °C, 50 °C, and 80 °C) on the capacitance of supercapacitors without the use of conducting carbon-black or a binder was studied.

## Graphical abstract

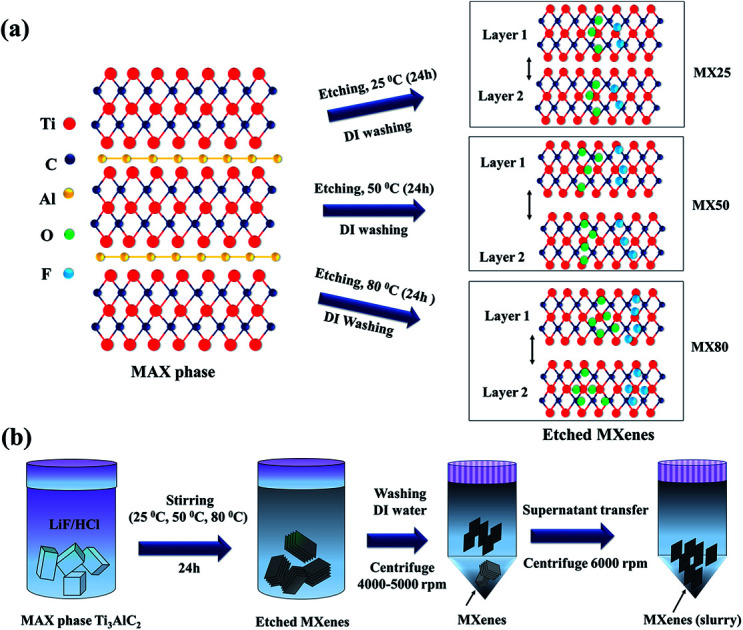

## Introduction

The layered inorganic 2D structures, MXenes, have been recently investigated widely in supercapacitor applications owing to their high surface area and conductivity.^1,2^ Numerous applications have been found including energy storage, 3D printing, fuel cells, EMI shielding, conductive inks, Li-ion batteries, and as oil additives.^2–11^ These materials are synthesized by the selective etching of MAX phases, generally represented by M_*n*+1_AX_*n*_, where M is an initial transition metal (Ti, V, Nb, *etc.*), A is a group 13 or 14 element in the periodic table and X denotes C and/or N atom, and *n* = 1, 2, or 3.^12^ After the selective etching of the ‘A’ element from MAX phases, the stacks of layered MXenes held together by van der Waals forces are described as M_*n*+1_X_*n*_T_*x*_, where T_*x*_ represents the terminal groups (*e.g.*, –OH, –O, –Cl or –F, *etc.*).^13,14^

Recently, Ti_3_C_2_T_*x*_ MXenes (T = O, F, OH, *etc.*) have been studied extensively in energy storage applications, including supercapacitors.^13,15,16^ However, the carbon-based materials lead to the electric double-layer capacitor (EDLC) behavior exhibiting low energy densities, and the MXenes exhibit a pseudo-capacitive behavior having higher energy densities due to redox reaction and intercalated faradaic reactions between the layers of MXene as well as at the surface.^17^ For the high capacitance in supercapacitors, the surface area is one of the most important parameters. Hence, the effective etching of MAX phases and complete exfoliation of MXene layers is highly desirable. To etch the MAX phases, strong acids, such as HF or LiF/HCl, are generally used.^15,18–22^ Besides these, etching by ammonium bifluoride (NH_4_HF_2_) and KOH has also been proposed.^23–25^ Among these etchants, the HCl/LiF etchant has been found to be the most suitable alternative to the highly toxic HF acid, and it is also used to synthesize more conducting clay-like MXenes. After etching, the MXene layers can be exfoliated further by sonication or by the metal ions-intercalation or guest molecules,^16,26,27^ fabricating hybrid structures with graphene.^28^

Another parameter that can affect the Al etching and forming MXene layers is the thermal treatment. In the case of HF acid-based etching for Ti_3_C_2_T_*x*_, there are studies detailing up to 65 °C in temperature.^29–31^ In the case of HCl/LiF, most of the etchings are performed to 45 °C,^15,32,33^ although there was a report on etching at 55 °C. It has been reported that increasing the etching temperature improves the exfoliation process, *i.e.*, from Ti_3_AlC_2_ to layered Ti_3_C_2_T_*x*_ MXenes, which also results in higher surface oxidation of the MXene layers after the extraction of Al.^21^ It has been observed that the delamination ratio increases with temperature first and then decreases.^21^ However, there has been no report for Ti_3_C_2_T_*x*_ MXene etching at higher temperatures (>60 °C) with the comparison of MXenes etched at different temperatures for energy storage applications.

Besides the etching temperature, another aspect in the Ti_3_C_2_T_*x*_ MXene is the etchant concentration. It had been earlier reported that high etchant concentrations (HF) lead to high surface areas and capacitances.^34^ MXene etching studies have previously focused on using 6 M or 9 M HCl/LiF etching acid combinations.^21,22^ Also, it was reported that the concentrated HCl (12 M) provided high quality MXene.^35^ Hence, in this study, we used concentrated 12 M HCl/LiF as an etching reagent to etch Ti_3_AlC_2_ at the elevated temperatures up to 80 °C for 24 h, and corresponding MXenes were used in fabricating the supercapacitors so as to analyze the effects of the etching temperature on the energy storage capacity of supercapacitors based on the MXenes without the use of carbon black and binder. To the best of our knowledge, there are no or negligible reports on the collective use of the concentrated HCl/LiF acid etching of MXenes at elevated temperatures and their comparative studies in supercapacitor applications without using carbon black and binder.

## Materials and methods

**Fig. 1:**
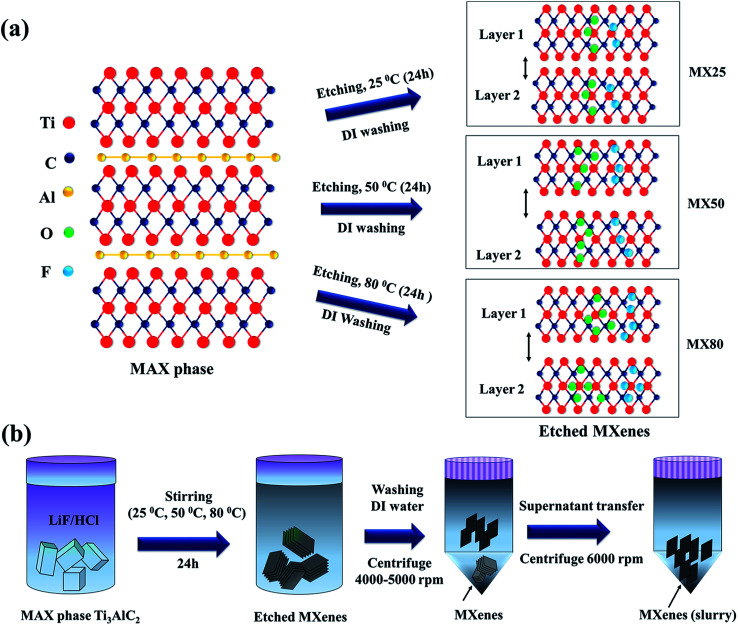
(a) Etching mechanism indicates that etching at higher temperatures causes higher exfoliation and number of functional groups on the MXene surface. (b) Ti_3_C_2_T_*x*_ MXene synthesis includes stirring and repeated centrifuging.

Ti_3_C_2_T_*x*_ MXenes were synthesized by selectively etching Ti_3_AlC_2_ [>98%, 200 mesh, Beijing Forsman Scientific Co., Ltd] using the HCl/LiF etchant, as reported earlier.^15^ In a typical process, 2 g LiF powder was mixed with 30 ml HCl (12 M) for 10 min. To this mixture, 1 g of Ti_3_AlC_2_ was added slowly and stirred for 24 h at different temperatures, *i.e.*, 25 °C, 50 °C, and 80 °C. The resultant mixtures were centrifuged at 4000 rpm and washed using DI water, and this was repeated until the pH was in the range of ∼5–6. The MXenes etched at 25 °C, 50 °C and 80 °C have been denoted as MX25, MX50, and MX80, respectively [Fig. 1(a)]. These MXenes were further sonicated in DI water for 30 min and centrifuged at 5000 rpm. The supernatant was transferred to another tube and again centrifuged at 6000 rpm to obtain the more exfoliated MXenes [Fig. 1(b)]. The precipitated MXene was used as slurry without adding carbon black and binder to fabricate the supercapacitors. This slurry was applied to Ni-foils with a thickness of ∼0.15 mm and area ∼ 3 × 3 cm^2^, and these plates were used as current collectors after drying at 100 °C for 12 h. The net weight of the active electrode material on both plates was determined by taking the difference of the weights before and after mass loading onto Ni-plates. The MXene-loaded supercapacitor plates, divided by a separator with a thickness of ∼25 μm (Celgard, USA) soaked in 1 M H_2_SO_4_ electrolyte, were assembled to form a two-electrode device.

The morphology and structure of the etched Ti_3_C_2_T_*x*_ MXenes were examined using a field emission scanning electron microscope (FESEM, SU8010 Hitachi, Japan) and Raman microscope (Renishaw, inVia, UK). The elemental composition of Ti_3_C_2_T_*x*_ has been confirmed *via* energy-dispersive X-ray (EDX) spectroscopy, and elemental distribution was determined *via* EDX mapping (EDX, Horiba). The composition and presence of the elements in the etched MXenes were also confirmed *via* X-ray photoelectron spectroscopy (XPS) (K-alpha (Thermo VG, U.K.)) using a monochromated X-ray source (Al Kα line: 1486.6 eV). The surface area and pore size distribution of the etched MXenes have been analyzed *via* the Brunauer–Emmett–Teller (BET) measurement technique (BELSORP-max, BEL Japan Inc.). The electrochemical studies were investigated *via* cyclic voltammetry (CV)/galvanostatic charge–discharge (CD) (Bio-logic, SP-150, USA) and electrochemical impedance spectroscopy (EIS) performed using potentiostat/galvanostat (Bio-logic, SP-200, USA). The MXene flake size estimations were performed *via* transmission electron microscopy (TEM) (JEM-F200, Jeol Ltd., Japan). The electrical conductivity was measured using a Hall measurement system (Ecopia, HMS-3000).

## Results and discussion

### Morphological and elemental analysis

**Fig. 2:**
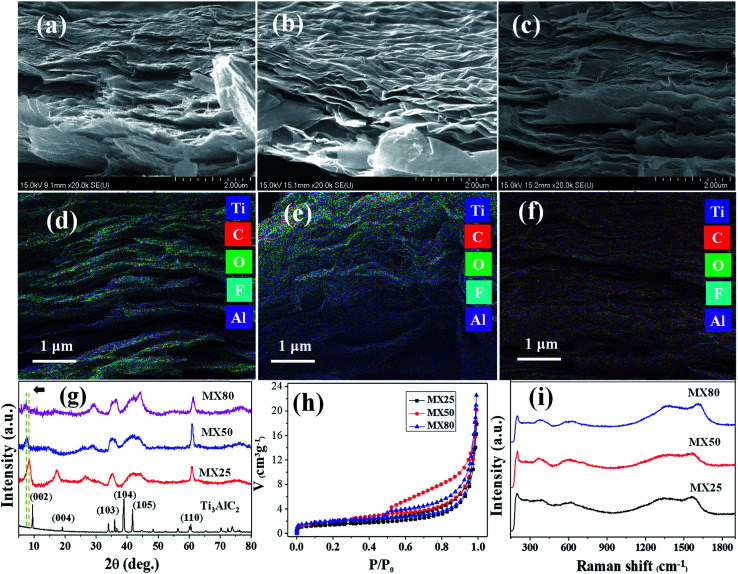
(a–c) FESEM images are shown for MX25, MX50, and MX80, respectively. (d–f) EDX maps with Ti, C, O, F, and Al are combined for MX25, MX50, and MX80, respectively. The colors are coded, as shown in the legends. (g) XRD spectra, (h) BET adsorption isotherms, and (i) Raman spectra of MX25, MX50, and MX80, are shown together.

The morphologies of MX25, MX50, and MX80 were investigated *via* FESEM, as shown in Fig. 2(a–c). The images reveal that the etched MXene layers have a thickness of <50 nm. It has been observed that all etched MXenes have stacked layers and the separations between layers for MX50 and MX80 appear to be greater than MX25. The higher temperature can help in activating the chemical reaction and quick removal of Al, and simultaneously the thermal vibrations help in segregating the adjacent layers. The distribution of the elements present in the different MXenes was confirmed by EDX mapping as, shown in Fig. 2(d–f). The EDS mappings indicate the uniform distribution of Ti, C, O, F, and Al elements.

The corresponding individual elemental maps indicating the distribution of Ti, C, O, F and Al elements in all MXenes are shown in Fig. S1(a–o) [ESI†], and the EDS spectra of MX25, MX50, and MX80 MXenes are shown in Fig. S1(p–r) [ESI†], respectively. The at% and wt% of MX25, MX50, and MX80 Ti_3_C_2_T_*x*_ MXenes are shown in Table S1 [ESI†]. It can be seen that the residual amount of Al in MX25 is 1.12 at%, and it reduces to 0.15 at% for MX80, indicating that the etching of Al has been improved with temperature. Also, it was observed that the oxygen content increases from ∼15 at% for MX25 to ∼29 at% for MX80, supporting that there is greater oxidation in MXene layers at high etching temperatures. The lateral size of MXene flakes, confirmed using FESEM and TEM, ranges between ∼300 nm and 1.5 μm with no significant size change depending on the etching temperature, as shown in Fig. S1A(a–f) [ESI†].

### XRD studies

The XRD patterns of MX25, MX50, and MX80 are shown along with that of the Ti_3_AlC_2_ phase in Fig. 2(g). However, the XRD pattern of the Ti_3_AlC_2_ MAX phase exhibits sharp peaks indexed to (002), (004), (103), (104), (105), and (106) (JCPDS 52-0875), and the peaks were reduced or disappeared in all MXenes indicating the removal of Al from Ti_3_AlC_2_. The (002) peak in the MAX phase shifted towards lower angles in all MXenes, indicating a widening of the gaps between the MXene-layers.^32^ It can be seen that the (002) and (004) peaks of MX50 shifted further towards lower angles as compared to those of MX25, indicating higher layer separation. In the case of MX80, however, the (002) and (004) peak intensities are very low, implying that the crystallinity of MX80 deteriorated. All of the etched MXenes show another characteristic peak (110) at 62.5°, confirming the formation of the Ti_3_C_2_ phase.^18^

### Surface area analysis

The specific surface area (SSA) of all MXenes was estimated using the BET measurement technique by nitrogen adsorption and desorption isotherm at 77 K. The SSA, as estimated from the BET plot [Fig. 2(h)] indicates a slight increment with an increase in temperature. The SSA of MX25 MXenes is ∼7.3 m^2^ g^−1^, whereas the SSA of MX50 and MX80 is ∼10.8 and ∼9.7 m^2^ g^−1^, respectively, indicating that the MXenes were exfoliated additionally by the etching at the higher temperature. However, the slight decrement in the SSA at 80 °C may be associated with partial adhesion between oxidized MXene layers,^21^ as also evident from the FESEM images. The pore size distribution, obtained using the BJH method (Barrett–Joyner–Halenda), was plotted for all MXenes, as shown in Fig. S1B [ESI†]. It was observed that all MXenes are microporous. However, MX25 shows a pore size distribution with a broad peak centered at 80 nm, and MX50 and MX80 exhibit monotonic increments without a peak, implying that the elevated temperature induced the disordered distribution of MXene flakes.

### Raman spectroscopic studies

The Raman spectra of MX25, MX50 and MX80 MXenes are shown in Fig. 2(i). It can be seen that all three MXenes have almost similar spectra, which indicate that MX25, MX50, and MX80 have similar chemical structures. The spectra exhibit the characteristic peaks at ∼200, ∼390, and ∼620 cm^−1^. The peak at ∼200 cm^−1^ can be associated with the Ti–C vibrations, whereas the peak ∼ 390 cm^−1^ is supposed to originate with the oxygen atom vibrations. The peak at ∼620 cm^−1^ can be related to the *E*_g_ vibrations of the carbon atoms in the Ti_3_C_2_T_*x*_ MXenes, which have –OH terminal groups.^36^ The two additional peaks centered at ∼1350 and ∼1580 cm^−1^ correspond to the D and G bands of the graphitic carbon, respectively.^37^ The defect related to the D band^38^ has higher intensity in case of MX80 as compared to the other counterparts, indicating that more defects are introduced during high temperature etching. Also, the reduced peak intensity at ∼200 cm^−1^ probably due to more oxidized MX80 can also be associated to nearby defects, resulting in weak out-of-plane vibrations.^39^

### XPS studies

**Fig. 3:**
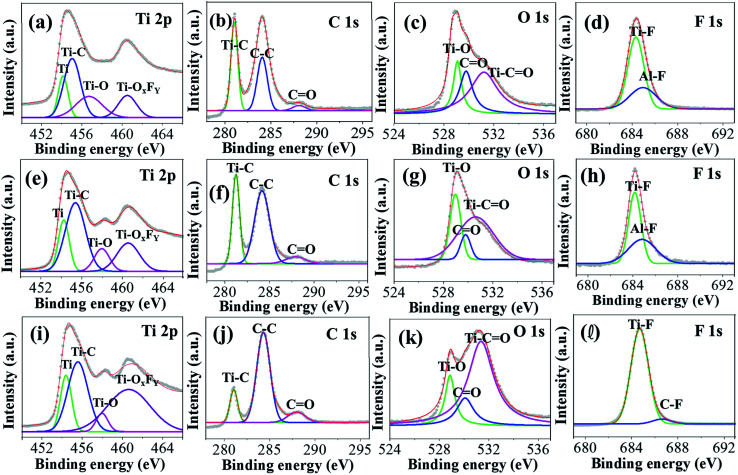
High-resolution XPS spectra of (a–d) MX25, (e–h) MX50, and (i–l) MX80 Ti_3_C_2_T_*x*_ MXenes corresponding to Ti 2p, C 1s, O 1s and F 1s peaks, respectively.

The elemental composition of the MX25, MX50, and MX80 MXenes was confirmed using an XPS analysis, and the corresponding high-resolution XPS spectra are shown in Fig. 3. Fig. S2(a–c) [ESI†] shows the combined XPS spectra of MX25, MX50, and MX80 MXenes, where all XPS spectra exhibit peaks corresponding to Ti 2p, C 1s, O 1s, F 1s, Cl 2p, and Al 2p. The peak at 454 eV due to Ti 2p_3/2_ indicates the metallic Ti(ii), whereas the peak at 455.7 eV indicates the Ti–C bond. Another peak at 460.8 eV may be due to either Ti–C or Ti–O_*x*_F_*y*_ in MXene [Fig. 3(a, e and i)].^40,41^ Similarly, in the C 1s plot, the peaks at 281.9 and 284.2 eV indicate the Ti–C bond and C–C bond, respectively [Fig. 3(b, f and j)].^40^ In the O 1s spectrum, the peaks at ∼529 and ∼530 eV indicate Ti–O and C=O bonds, respectively, whereas the 531 eV peak may exhibit a metal-carbonate character, possibly the Ti–C=O group [Fig. 3(c, g and k)] and may be associated with the O terminal groups.^41^ Particularly, high areas of O 1s peaks in MX80 may indicate the high oxygen content. The Ti–C=O binding is enhanced at 80 °C etching is probably due to the emergence of more oxygen based functional groups at the MXene surface. In the F 1s plot, the peaks at ∼684–685 eV for MX25 and MX50 indicate a metal-fluoride bond [Fig. 3(d and h)], which may be a Ti–F or Al–F bond, indicating F as the terminal group on Ti_3_C_2_. However, in the case of MX80, a C–F peak is observed, instead of Al–F exhibiting an organic-fluoride peak at ∼687 eV, which may be due further etching of Al [Fig. 3(l)].

The atomic% of all elements present in all MXenes obtained from XPS are shown in Table S2.† The trend of higher oxygen content in MXenes etched at higher temperatures, *i.e.* MX80, accords well with that observed in EDS, as shown in Fig. S2(d) [ESI†]. Besides these, small amounts of Li, Cl, and Al were also observed. It can be seen that the Al etching improves slightly with temperature, but the terminal groups such as Li and Cl are not reduced due to additional oxidation.

### Cyclic voltammetry (CV) curves

**Fig. 4:**
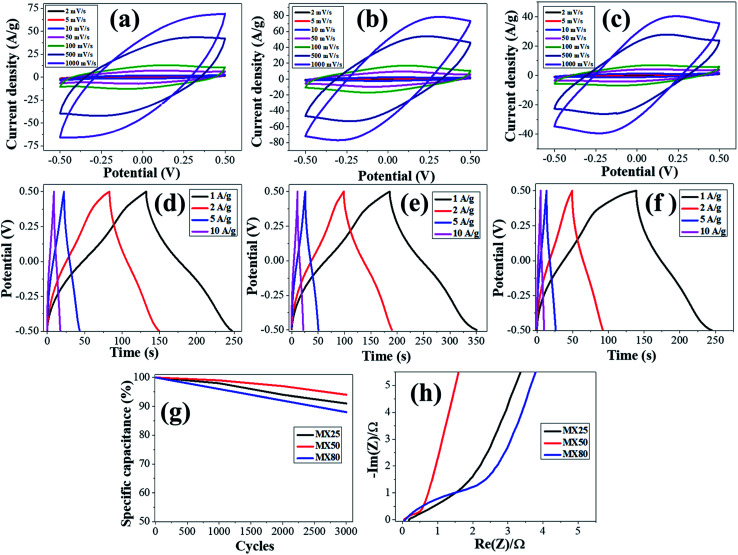
Electrochemical characteristics of supercapacitors: (a–c) CV curves, (d–f) CD curves, (g) cyclic stability up to 3000 cycles at the scan rate of 1000 mV s^−1^, and (h) Nyquist plots, for MX25, MX50, and MX80 MXenes, respectively.

The electrochemical characteristics of MX25-, MX50-, and MX80-based supercapacitors are shown in Fig. 4, and the capacitances were estimated using CV plots in −0.5 to 0.5 V at 2 to 1000 mV s^−1^ scan rates. The CV plots exhibit quasi-rectangular shapes in case of MX25 supercapacitors [Fig. 4(a)]. However, as the temperature is increased slight redox peaks start appearing in CV plots of the MX50- and MX80-based supercapacitors [Fig. 4(b and c)]. The appearance of a redox peak in the rectangular shape can be associated with the emergence of pseudo-capacitance in addition to the electric double layer capacitance character. The onset of faradaic or redox peak may be associated to oxygen-containing functional groups.^42,43^ The specific *C*_g_ of the supercapacitors was calculated using the following relation:
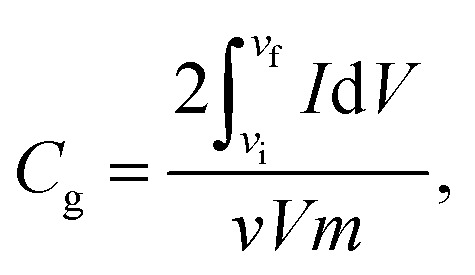
where 
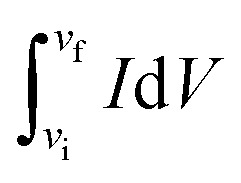
 is the area of the CV loop with *v*_i_ as initial and *v*_f_ as final voltages; *v*, *V*, and *m* are the scan rate, voltage scan window, and the total weight of active electrode material on both electrodes, respectively.

The CV curve-based specific capacitances for the MX25, MX50, and MX80 were found 581, 657, 421 F g^−1^ at the scan rate of 2 mV s^−1^, respectively. These values are significant considering that no binder or conductive carbon-black was mixed in formulating the slurry. As the surface area values of all MXenes are not high, the main contribution in capacitance is attributed to the pseudo-capacitance character, instead of the EDLC behavior in all supercapacitors. It has been reported earlier that the capacitance is attributed to the contribution from redox capacitance in the MXene-based supercapacitors having acidic electrolytes. The redox reaction involves the electrochemical adsorption of ions on the surface of MXene corresponding to charge-transfer. The high capacitance is due to the occurrence of intercalated faradaic reactions within the layers as well as at the surface of MXene.^17^*C*_g_ increases from MX25 to MX50 and then reduces for MX80 MXene. The higher capacitance for MX50 may be associated with the higher surface area of MX50 MXenes as compared to those of MX25 and MX80 MXenes. The possible reason for the reduced *C*_g_ for MX80 is that at higher etching temperatures, the excessive oxide layer with the terminal groups prevents the double layer formation on the MXene surface, as evident from EDS and XPS spectra. Also, the defects can lead to the reduction of the active sites accountable for the pseudo-capacitive redox reactions, which can lead to the reduced capacitance.^44^ Similar trends are observed at higher scan rates, as shown in Table 1. The oxygen based functional groups have impacts on the electrochemical properties of the materials. These functional groups reduce the electrode surface conductivity and also restrict the migration of ions.^45^ It has also been reported earlier that Ti_3_C_2_ MXene with terminal oxygen groups shrink during the electrochemical reaction.^17^ Also, the MX50 MXene maintains the highest cyclic stability up to (>90%) up to 3000 cycles at a higher scan rate of 1000 mV s^−1^, indicating that the supercapacitors based on MXene etched at 50 °C can sustain its high capacitance even at high scan rates [Fig. 4(g)]. After 3000 cycles, the capacitance retention values of MX25-, MX50-, and MX80-based supercapacitors are ∼91%, ∼94%, and ∼88%, respectively.

**Table 1:** Specific capacitance for the supercapacitors based on MXenes measured at different scan rates

Scan rate (mV s^−1^)	Specific capacitance (F g^−1^) MX25	Specific capacitance (F g^−1^) MX50	Specific capacitance (F g^−1^) MX80
5	539	612	396
10	498	564	345
50	426	493	277
100	377	424	221
500	197	238	136
1000	121	136	79

### Charge–discharge (CD) studies

The charge–discharge characteristics of MX25, MX50, and MX80 MXene based supercapacitors are studied from CD curves obtained at various current densities, 1 A g^−1^, 2 A g^−1^, 5 A g^−1^ and 10 A g^−1^ within the −0.5 to 0.5 V voltage range. These CD plots, shown in Fig. 4(d–f), indicate no voltage-drop in all curves studied up to a 10 A g^−1^ current density. This means that the supercapacitors exhibit a fast charge–discharge conversion behavior with low series resistances. The deviation of CD curves from a triangular shape may be due to the pseudo-capacitive behavior instead of the EDLC behavior. The low voltage drop (or IR loss) may also be associated with higher electrical conductivity of MXene electrodes. The electrical conductivity, measured using the van der Pauw method, indicates that MX80 MXene has lower conductivity than the others [Table S3, ESI†]. The lower electrical conductivity of MX80 can be attributed to the presence of oxygen based functional groups and defects. However, the electrical conductivity of MX25 and MX50 are not much different probably due to lower oxidation and low defects. The coulombic efficiency appears less than 100% in all cases; however, MX50 MXene has highest coulombic efficiency as compared to the rest of supercapacitors. In the case of MX80 MXene at lower current densities, the CD curve is nonlinear and asymmetric between the charging and discharging region, which means that the oxidized MXene accompanies unintended electrochemical reactions. It has been reported earlier that the oxidation of Ti_3_C_2_T_*x*_ may lead to its degradation.^46,47^

### Electrochemical impedance spectroscopy and Nyquist plots

The electrochemical performances of MX25, MX50, and MX80 MXene based supercapacitors were estimated using EIS measurements with Nyquist impedance plots obtained at a 10 mV sinusoidal signal in the frequency range from 100 kHz to 0.1 Hz [Fig. 4(h)]. Nearly, vertical lines in all Nyquist plots at lower frequencies indicate the capacitive character of all electrodes,^48^ and low IR losses may also be associated with higher electrical conductivity of MXene electrodes. Based on the Randles equivalent circuit, all supercapacitors are analyzed, as shown in Fig. S3 and Table S4 [ESI†]. It was observed that all the supercapacitors have low series resistance (*R*_s_). However, the charge-transfer resistance (*R*_ct_) increases as the MXene etching temperature is increased. Particularly, MX80 shows a slight hint of a semicircle at the high-frequency region, corresponding to *R*_ct_. Furthermore, MX25- and MX50-based supercapacitors have low Warburg impedance (*W*) as compared to MX80-based supercapacitors, as shown in Fig. S3.† A higher number of functional groups at the surface of the oxidized MXene may lead to the resistance to the diffusion of the ions near the MXene surface.

The values of *R*_s_, *R*_ct_, *W*, and *C*_1_ as derived from fitting to Randles circuit are 0.11 Ω, 0.45 Ω, 4.3 Ω, 467 F g^−1^; 0.12 Ω, 0.4 Ω, 1.75 Ω, 600 F g^−1^; and 0.12 Ω, 1.4 Ω, 3.3 Ω, 388 F g^−1^ for MX25, MX50, and MX80 MXenes, respectively, as shown in Table S4 [ESI†]. The values of *C*_1_ are close to those obtained using CV plots. The higher *R*_ct_ and *W* values in MX80 as compared to MX50 and MX25 may be ascribed to functional groups at these MXene surfaces.

It is worthy of notice that the high performance of the supercapacitors was achieved without the use of carbon black and binder. Many earlier reports on MXene supercapacitors used conductive carbon to create conducting networks between the MXene sheets.^16,44,49,50^ It was believed that the addition of the activated carbon caused higher capacitance and lower IR loss due to high surface area and conductivity of carbon materials.^51^ In addition, the carbon-based materials can introduce the EDLC behavior to the supercapacitors forming hybrid supercapacitors. However, the EDLC capacitance contribution of carbon is minor as compared to the pseudo-capacitance of MXene.^52^ Furthermore, as an alternate, MXene itself can also act as a conductive additive due to its high conductivity and flexibility.^53^

## Conclusions

In this study, two strategies were adopted to improve the supercapacitor properties using Ti_3_C_2_T_*x*_ MXene without the use of carbon black or binder. First, the etching acid (HCl) concentration is higher (12 M), and second, the studies have been optimized at different etching temperatures. It was concluded that the etching at high temperature leads to high surface area and specific capacitance as thermal vibration assists the exfoliation of MXene layers. However, excessively elevated temperatures may degrade the quality of MXene due to oxidized groups, resulting in lowering specific capacitance. Hence, MXene etched at the intermediate temperature (50 °C) was found suitable for supercapacitor applications. The specific capacitance of Ti_3_C_2_T_*x*_-based supercapacitors increased from 581 F g^−1^ for MXenes etched at 25 °C to 657 F g^−1^ for those etched at 50 °C at 2 mV s^−1^ scan rate; however, it reduces to 421 F g^−1^ as the etching temperature was increased to 80 °C. The MX50 MXene-based supercapacitor exhibits higher cyclic stability up to 3000 cycles at 1000 mV s^−1^ scan rate, and their CD curves are more symmetric as compared to MX25 and MX80 MXenes. EIS studies also indicate the superior performance of MX50-based supercapacitors in terms of charge-transfer resistance, Warburg impedance, and capacitance. This study can provide insights into both MXene etching at optimized temperatures as well their use in supercapacitors even without using carbon black or binder.

## Author contributions

Research design, S. K. and Y. S.; experiments and data analysis, S. K., D. K., H. H., Y. L., and Y. S.; paper writing, S. K., N. L., and Y. S.

## Conflicts of interest

The authors declare no conflicts of interest.

## Supplementary Material

RA-010-D0RA05376G-s001
