# Supplementary material for: Effect of Ti3C2Tx MXenes etched at elevated temperatures using concentrated acid on binder-free supercapacitors
Source: RSC Adv. 2020 Nov 17;10(68):41837–45. doi: 10.1039/d0ra05376g (PMC9057861; doi:10.1039/d0ra05376g)
Supplement: RA-010-D0RA05376G-s001 [file RA-010-D0RA05376G-s001.pdf]

Electronic Supplementary Material (ESI) for RSC Advances.
This journal is © The Royal Society of Chemistry 2020

Supplementary information

Effect of $Ti_3C_2T_x$ MXenes Etching at Elevated Temperatures using Concentrated Acid on Binder-free Supercapacitors

Sunil Kumar[1,2], Dongwoon Kang[1], Hyeryeon Hong[1], Malik Abdul Rehman[3], Yeon-jae Lee[1], Naesung Lee[1,2], Yongho Seo[1,2*]

[1]Department of Nanotechnology and Advanced Materials Engineering, Sejong University, Seoul, 05006, South Korea

[2] HMC, Sejong University, Seoul, 05006, South Korea

[3]Department of Materials Science and Engineering, Yonsei University, Seoul, 03722, South Korea

[*]Corresponding author: Email- yseo@sejong.ac.kr

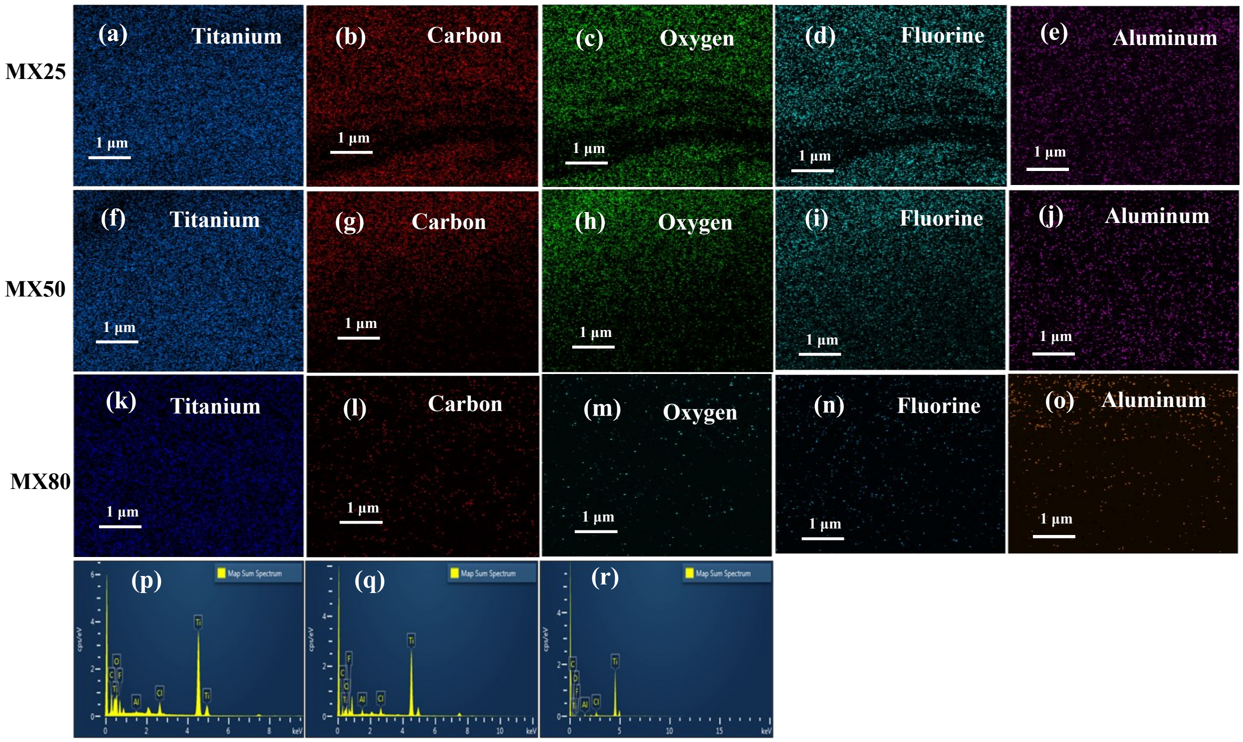

Figure S1. EDX maps of (a-e) Ti, C, O, F and Al in MX25, (f-j) Ti, C, O, F and Al in MX50, (k-o) Ti, C, O, F and Al in MX80, and (p-r) EDX spectra of MX25, MX50 and MX80 MXenes, respectively.

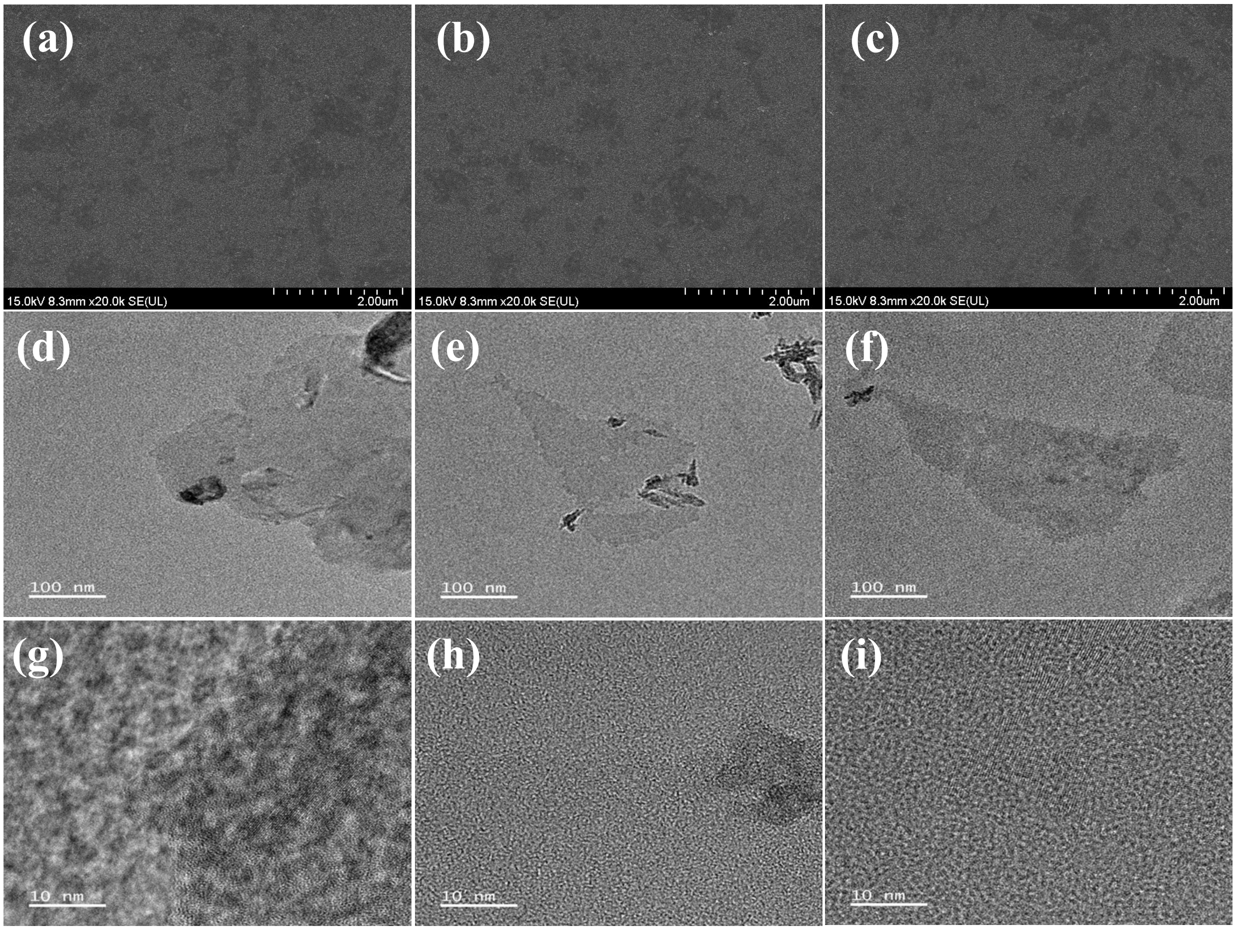

Figure S1A. (a-c) FESEM images, (d-f) Low magnification TEM images, and (g-i) HRTEM images of MX25, MX50, and MX80 MXenes, respectively.

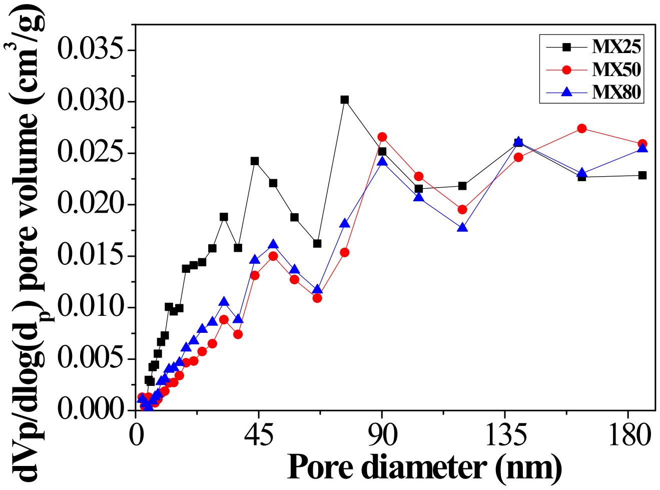

Figure S1B. Pore size distribution (BJH plots) of MX25, MX50, and MX80 MXenes.

Table S1. EDX elements in MX25, MX50, and MX80 MXenes

**Element**	MX25		MX50		MX80	
	**Wt.%**	**Atomic %**	**Wt.%**	**Atomic %**	**Wt.%**	**Atomic %**
**Ti**	51.95	43.58	48.06	31.32	34.34	13.96
**C**	25.59	37.67	21.97	34.29	25.25	40.95
**O**	12.09	12.86	15.79	18.81	24.18	29.44
**F**	6.76	2.68	11.51	13.43	14.06	14.42
**Cl**	2.57	2.10	1.93	1.20	1.96	1.07
**Al**	1.04	1.12	0.74	0.94	0.20	0.15
**Total**	100.00	100.00	100.00	100.00	100.00	100.00

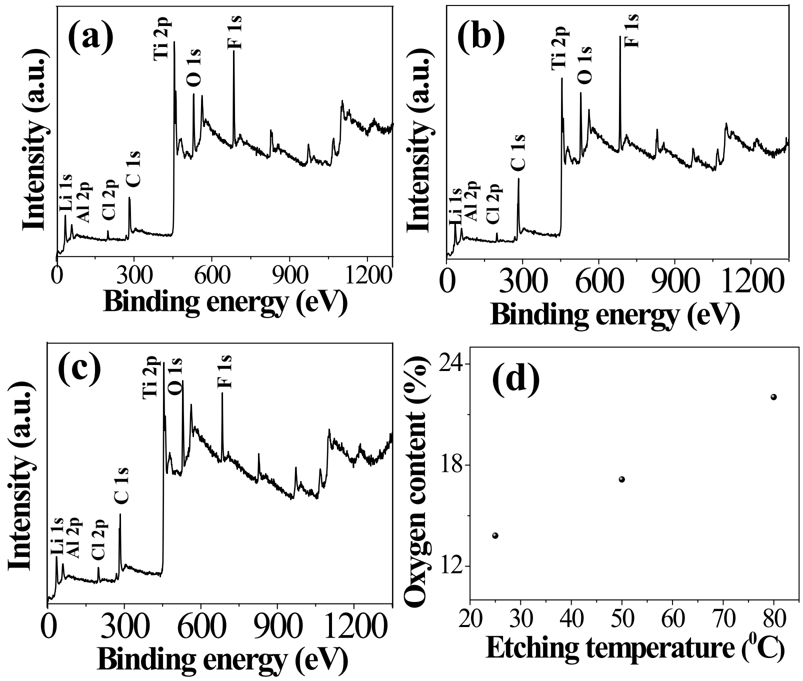

Figure S2. Complete XPS spectra of (a) MX25, (b) MX50, (c) MX80 MXenes, and (d) XPS based oxygen content in MXenes at different etching temperatures

Table S2. XPS elemental composition of MX25, MX50, and MX80 MXenes

Element	*MX25*	*MX50*	*MX80*
Ti 2p	28.94	29.26	29.60
C 1s	35.17	29.88	32.11
O 1s	13.81	17.15	22.03
F 1s	17.88	20.86	15.51
Li 1s	2.66	2.03	1.40
Cl 2p	0.76	0.55	0.95
Al 2p	0.78	0.27	0.20
**Total**	100.00	100.00	100.00

Electrode	R_s (Ω)	R_{ct} (Ω)	C_1 (F)	C_2 (F)	W (Ω)	Mass (mg)	C_1/g (F/g)

Table S3. Conductivity of MX25, MX50 and MX80 MXenes using van der Pauw Measurement System

MXene	MX25	MX50	MX80
Conductivity (S/m)	8.439×10^3	10.440×10^3	4.154×10^3

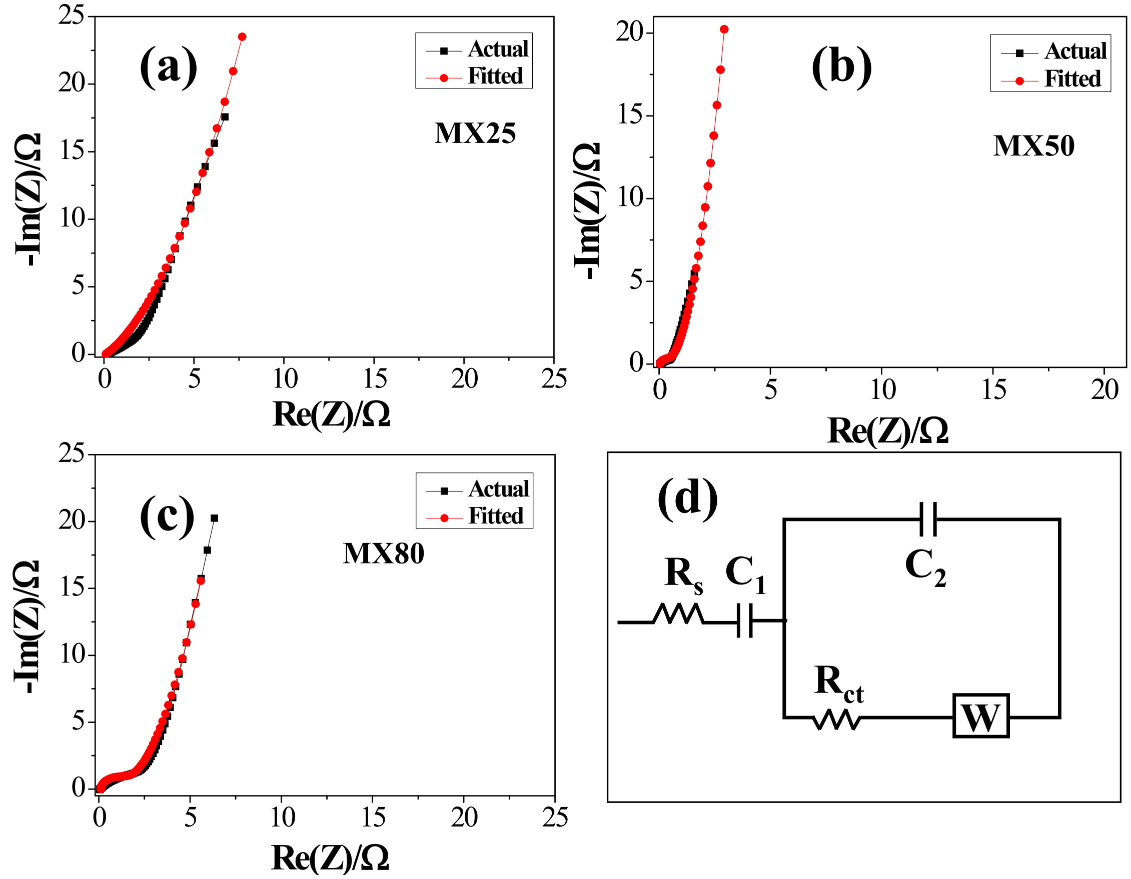

Figure S3. (a-c) Software fitted Nyquist plots of MX25, MX50, and MX80 MXenes, respectively, and (d) Randles equivalent circuit

MX25 MXene	0.11	0.45	0.98	0.0055	4.3	2.1	467
MX50 MXene	0.11	0.034	0.84	0.0065	1.75	1.4	600
MX80 MXene	0.12	1.4	0.62	0.0033	3.3	1.6	388

Table S4. Fitting results for the equivalent circuit to Nyquist plots based EIS parameters
